# 固相萃取-液相色谱-串联质谱法测定污水中8种新烟碱类农药

**DOI:** 10.3724/SP.J.1123.2023.11010

**Published:** 2024-09-08

**Authors:** Haitang WANG, Hanyin LI, Qiwei LU, Shilong HE

**Affiliations:** 1.中国矿业大学环境与测绘学院,江苏徐州 221116; 1. School of Environment and Spatial Informatics, China University of Mining and Technology, Xuzhou 221116, China; 2.江苏省徐州环境监测中心, 江苏徐州 221000; 2. Xuzhou Environmental Monitoring Center of Jiangsu, Xuzhou 221000, China; 3.徐州高新技术产业开发区,江苏徐州 221000; 3. Xuzhou High-tech Industrial Development Zone, Xuzhou 221000, China

**Keywords:** 液相色谱-串联质谱, 固相萃取, 新烟碱类农药, 污水, 基质效应, 响应曲面法, liquid chromatography-tandem mass spectrometry (LC-MS/MS), solid phase extraction (SPE), neonicotinoid pesticides, wastewater, matrix effect, response surface methodology (RSM)

## Abstract

新烟碱类农药作为一类新型农药,由于其对非靶标生物造成生态风险而引起广泛关注。为了实现污水中痕量新烟碱类农药的快速、准确定量,本研究建立了同时检测污水中8种新烟碱类农药(呋虫胺、*E*-烯啶虫胺、噻虫嗪、噻虫胺、吡虫啉、氯噻啉、啶虫脒和噻虫啉)的固相萃取-液相色谱-串联质谱法。确定选择色谱流动相类型和质谱参数后,采用单因素法确定固相萃取(SPE)的条件:萃取柱类型为HLB (500 mg/6 mL),上样体积为500 mL,上样速度为10 mL/min,样品pH为6~8。通过优化色谱梯度洗脱程序、样品的稀释倍数并采用同位素内标定量法降低污水样品的基质效应,确定污水稀释5倍进行前处理,采用ZORBAX Eclipse Plus C18色谱柱(100 mm×2.1 mm, 1.8 μm),以含0.1%甲酸的2 mmol/L乙酸铵水溶液和甲醇为流动相进行梯度洗脱,在正离子多反应监测模式下分析10 min,用吡虫啉-d4作为同位素内标进行定量。通过响应曲面法进一步优化SPE的淋洗液及洗脱液类型和用量,确定用10%甲醇水溶液淋洗,7 mL甲醇-乙腈(1∶1, v/v)混合溶液洗脱。8种新烟碱类化合物在相应范围内线性关系良好(线性相关系数(*r*)均大于0.9990),方法检出限(MDL)为0.2~1.2 ng/L,方法定量限(MQL)为0.8~4.8 ng/L,在低、中、高3个加标水平下的加标回收率为82.6%~94.2%, RSD为3.9%~9.4%。该方法成功用于4个城镇污水处理厂进水水样的分析,8种新烟碱类农药的检出含量为ND~256 ng/L。与类似方法相比,该方法检出限低,准确度高,适用于污水中8种新烟碱类农药的痕量检测。

## Abstract (en)

Neonicotinoid pesticides are a relatively new class of pesticides that have garnered significant attention owing to their potential ecological risks to nontarget organisms. A method combining solid phase extraction with liquid chromatography-tandem mass spectrometry (SPE-LC-MS/MS) was developed for the rapid and accurate detection of eight neonicotinoid pesticides (dinotefuran, *E*-nitenpyram, thiamethoxam, clothianidin, imidacloprid, imidaclothiz, acetamiprid, and thiacloprid) in wastewater. The chromatographic mobile phase and MS parameters were selected, and a single-factor method was used to determine the optimal column type, extraction volume, sample loading speed, and pH for SPE. The optimal parameters were as follows: column type, HLB column (500 mg/6 mL); sample extraction volume, 500 mL; sample loading speed, 10 mL/min; and sample pH, 6-8. The matrix effects of the wastewater samples were reduced by optimizing the chromatographic gradient-elution program, examining the dilution factor of the samples, and using the isotope internal standard calibration method. Prior to analysis, the wastewater samples were diluted 5-fold with ultrapure water for pretreatment. Subsequently, 2 mmol/L ammonium acetate aqueous solution containing 0.1% (v/v) formic acid and methanol was used as mobile phases for gradient elution on a ZORBAX Eclipse Plus C18 column (100 mm×2.1 mm, 1.8 μm). The samples were quantified using positive-ion multiple reaction monitoring (MRM) mode for 10 min. Imidacloprid-d4 was used as the isotope internal standard. The SPE process was further optimized by applying response surface methodology to select the type and mass of rinsing and elution solvents. The optimal pretreatment of the SPE column included rinsing with 10% methanol aqueous solution and elution with methanol-acetonitrile (1∶1, v/v) mixture (7 mL). The eight neonicotinoid pesticides showed satisfactory linearity within the relevant range, with linear correlation coefficients (*r*) all greater than 0.9990. The method detection limits (MDLs) ranged from 0.2 to 1.2 ng/L, and the method quantification limits (MQLs) ranged from 0.8 to 4.8 ng/L. The average recoveries of the eight neonicotinoid pesticides were in the range of 82.6%-94.2% at three spiked levels, with relative standard deviations (RSDs) ranging from 3.9% to 9.4%. Finally, the optimized method was successfully applied to analyze wastewater samples collected from four sewage treatment plants. The results indicated that the eight neonicotinoid pesticides could be generally detected at concentrations ranging from not detected (ND) to 256 ng/L. The developed method has a low MDL and high accuracy, rendering it a suitable choice for the trace detection of the eight neonicotinoid pesticides in wastewater when compared with other similar methods. The proposed method can be utilized to monitor the environmental impact and assess the potential risks of neonicotinoid pesticides in wastewater, thus promoting the protection of nontarget organisms and the sustainable use of these pesticides in agriculture.

新烟碱类化合物因具有高效、广谱和高选择性及对哺乳动物低毒等优势在全球范围内被广泛使用^[1]^。使用最普遍的新烟碱类化合物主要有8种,分别是呋虫胺(dinotefuran)、*E*-烯啶虫胺(*E*-nitenpyram)、噻虫嗪(thiamethoxam)、噻虫胺(clothianidin)、吡虫啉(imidacloprid)、氯噻啉(imidaclothiz)、啶虫脒(acetamiprid)和噻虫啉(thiacloprid)。由于用量大、挥发性低、水溶性高以及半衰期长,新烟碱类化合物广泛存在于水、土壤和空气,甚至人体中,具有较大的环境风险^[2]^。研究显示新烟碱类农药对非靶标生物影响较大^[3]^,如降低蜂群密度,导致蝴蝶发育停滞和鸟类多样性减少^[4,5]^,以及对人类细胞系中的免疫信号传导产生不利影响^[6]^。环境中的新烟碱类化合物除来自农药施用外,污水处理厂由于传统的污水处理单元对新烟碱类农药的去除率低^[6]^,也成为主要污染源之一,因此准确定量分析污水中新烟碱类农药意义重大。

水体中新烟碱类农药的前处理方法主要为固相萃取(SPE)法,由于其极性大、蒸汽压低、辛醇-水分配系数值低及在环境中含量普遍较低等特点,分析方法以液相色谱法(LC)和液相色谱-串联质谱法(LC-MS/MS)为主。LC检测灵敏度相对较低,定性能力较差,而LC-MS/MS具有高灵敏度、高通量以及假阳性率较低等优点,更适用于复杂基质中新烟碱类农药的痕量分析^[7]^。LC-MS/MS最常用的两种电离方式是电喷雾电离(ESI)和大气压化学电离(APCI),而ESI模式更适用于极性较大的新烟碱类农药的分析测定^[8]^,但在使用该方法分析城镇污水中低浓度新烟碱类农药时,基质效应(ME)会影响测定精度、选择性和灵敏度^[9]^。目前很多学者对于降低水果、蔬菜、尿液和血浆中的基质效应进行研究^[10]^,但在分析污水中新烟碱类农药的相关研究中,降低基质效应的报道还相对较少。

减少基质效应的方法主要包括优化色谱条件和样品制备方法^[11]^、减少进入系统的样品量或者对样品进行稀释后进样^[12]^、使用同位素内标定量^[13]^、采用基质匹配标准曲线定量^[14]^等。Ferrer等^[12]^的研究表明稀释因子为5时,足以消除大部分基质效应,但会损失分析灵敏度。Kloepfer等^[13]^指出同位素内标价格昂贵且不易获得,而用基质匹配标准曲线定量需要大量空白提取物,为每个单独的基质源准备校准标准品和质量控制标准品是不切实际的。因此在许多情况下,可以选择几种方法共同使用以满足定量要求。

本工作旨在建立城镇污水中8种新烟碱类化合物的SPE-LC-MS/MS准确定量分析方法。本工作通过优化色谱和质谱参数,单因素优化萃取柱类型、上样体积、上样速度、样品pH值,响应曲面法考察淋洗液、洗脱液和洗脱体积,确定方法的最佳参数;并阐明降低污水中基质效应的方法,评估该方法的检出限、精密度和正确度,为污水中新烟碱类农药的准确定量提供参考。

## 1 实验部分

### 1.1 仪器、试剂与材料

1290-6460液相色谱-串联质谱仪(美国Agilent公司); Aspe-899全自动固相萃取仪(日本GL Science公司); Oasis HLB固相萃取柱(500 mg/6 mL)、一次性无菌注射器(5 mL)和聚四氟乙烯滤膜(0.22 μm、0.45 μm,美国Waters公司)。

甲醇和乙腈(色谱纯)购于美国TEDIA公司;甲酸和乙酸铵(优级纯)购于美国Merck公司;纯净水购于屈臣氏公司。8种新烟碱类标准品和吡虫啉-d4(纯度98%)均购自德国Dr. Ehrenstorfer公司。

准确称取10. 00 mg(精确到0. 01 mg)8种新烟碱类标准品,置于10 mL棕色容量瓶中,用甲醇溶解并定容至刻度,配成质量浓度为1. 00 mg/mL的单标准储备溶液,于-20 ℃冰箱中保存。分别准确移取0.1 mL上述单标准储备液于10 mL容量瓶中,混匀后用甲醇定容至刻度,配制成质量浓度为10 μg/mL的中间混合标准使用溶液,于-20 ℃冰箱中保存。使用时用甲醇稀释成所需工作浓度。

### 1.2 样品前处理

将水样在负压条件下通过0.45 μm聚四氟乙烯滤膜后避光储存在棕色玻璃瓶中,取100 mL水样用纯水稀释至500 mL,用全自动固相萃取法进行分离富集,具体步骤如下:分别用10 mL甲醇、10 mL水以5 mL/min流速活化固相萃取柱,以10 mL/min速度上样500 mL后用10 mL 10%甲醇水溶液淋洗萃取柱,氮吹30 min,然后用7 mL甲醇-乙腈(1∶1, v/v)以2 mL/min速度进行洗脱,将洗脱液在35 ℃下浓缩至体积小于1 mL,再用甲醇-乙腈(1∶1, v/v)定容至1 mL,加入内标吡虫啉-d4使其质量浓度为50 μg/L,用0.22 μm的滤膜过滤收集,待上机分析。

### 1.3 分析条件

#### 1.3.1 色谱条件

色谱柱:Agilent ZORBAX Eclipse Plus C18柱(100 mm×2.1 mm, 1.8 μm);流动相:含0.1%甲酸的2 mmol/L乙酸铵水溶液(A)和甲醇(B);流速:0.3 mL/min;进样体积:2 μL;柱温:40 ℃;梯度洗脱条件:0~5 min, 10%B~45%B; 5~9 min, 45%B~80%B; 9~9.1 min, 80%B~10%B, 9.1~10.1 min, 10%B。

#### 1.3.2 质谱条件

电喷雾离子源,正离子模式(ESI^+^);离子源喷雾电压:3500 V;离子源温度:300 ℃;雾化气压力:0.207 MPa;干燥气温度:325 ℃;鞘气温度:350 ℃;干燥气流速:6 L/min;鞘气流速:11 L/min。各目标化合物及内标物的质谱参数见表1。

**表1:** 目标化合物的保留时间和质谱参数

Compound	Retention time/min	Precursor ion (*m/z*)	Product ion (*m/z*)	Fragmentor/V	Collision energy/eV
Dinotefuran	3.35	203.2	129.1^*^	80	12
			87.1	80	14
*E*-Nitenpyram	3.87	271.1	99.1	95	14
			56.2^*^	95	23
Thiamethoxam	5.07	292.1	211.0^*^	80	10
			131.8	80	18
Clothianidin	5.37	250.1	168.9^*^	85	14
			131.8	85	16
Imidacloprid-d4	6.09	260.1	213.1^*^	100	14
			179.1	100	20
Imidacloprid	6.11	256.1	209.1^*^	85	12
			175.1	85	18
Imidaclothiz	6.26	262.1	181.0^*^	95	16
			122.0	95	35
Acetamiprid	6.72	223.1	126.0^*^	110	20
			56.1	110	16
Thiacloprid	7.45	253.1	125.9^*^	110	20
			89.9	110	45

* Quantitative ion.

## 2 结果与讨论

### 2.1 仪器条件优化

#### 2.1.1 色谱条件的优化

流动相的选择直接影响各目标化合物的色谱分离效果及质谱离子化效率。常用甲醇和乙腈作为新烟碱类农药分析中流动相的有机组分^[15]^,在流动相中加入适当的有机酸或缓冲盐可以提高目标化合物的电离效率,提高分析方法的灵敏度^[16]^。实验考察了水-乙腈、水-甲醇、0.1%甲酸水-甲醇、含0.1%甲酸的2 mmol/L乙酸铵水溶液-甲醇、含0.1%甲酸的5 mmol/L乙酸铵水溶液-甲醇5种流动相对目标化合物离子化效率的影响。结果如图1所示,用含0.1%甲酸水的2 mmol/L乙酸铵水溶液-甲醇和含0.1%甲酸的5 mmol/L乙酸铵水溶液-甲醇作流动相时,目标化合物的响应值均较高,且相差不大,说明加入甲酸和乙酸铵有利于[M+H]^+^加合形式的稳定。考虑到对环境的影响,选择含0.1%甲酸的2 mmol/L乙酸铵水溶液-甲醇作为流动相。

**图1:**
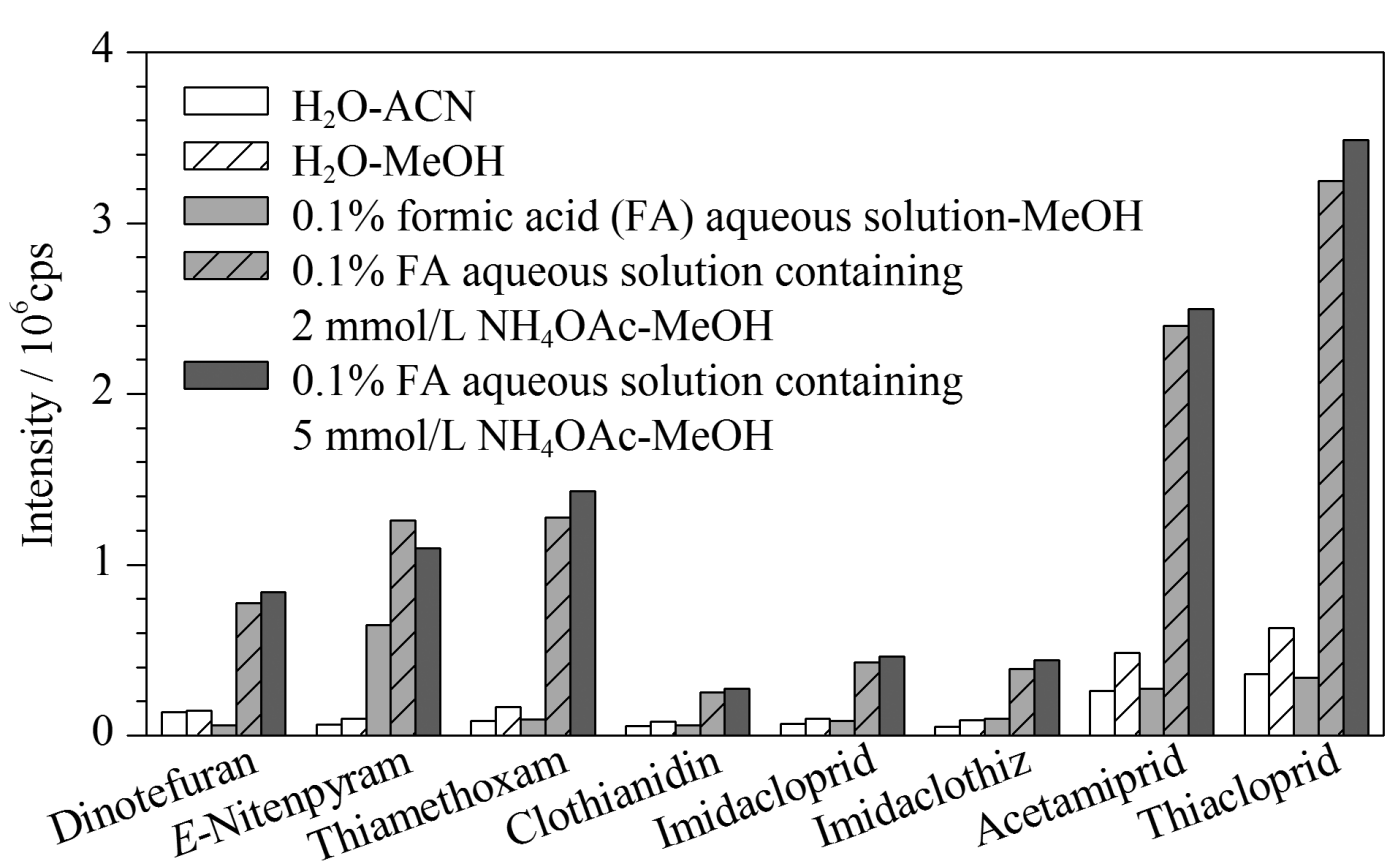
不同流动相下目标化合物的响应值

#### 2.1.2 质谱条件的优化

配制质量浓度为1.0 mg/L的各目标化合物的混合标准溶液,对目标化合物及内标物的质谱条件进行优化,包括母离子、子离子、碎裂电压、碰撞能量(CE)等参数。在全扫描(Scan)模式下对新烟碱类化合物进行一级质谱分析,确定每种新烟碱类化合物的母离子;再通过对每种化合物的母离子进行二级质谱扫描优化碎裂电压;在优化后的碎裂电压下通过子离子扫描模式确定每种化合物的碎片离子信息,并对其碰撞能量进行优化,使新烟碱类化合物特征离子与其子离子碎片的离子强度达到最大,具体参数见表1。

### 2.2 前处理条件的单因素法优化

在污水中8种新烟碱类农药检测方法的建立初期,为了避免基质效应对回收率的影响,选用超纯水作为基质进行加标试验。以回收率(提取前加标的峰面积/纯标准溶液的峰面积)作为评价指标,使用外标法定量,对固相萃取柱类型、上样体积、上样速度、样品pH值等参数通过单因素法进行优化,确定较合理的前处理条件。

#### 2.2.1 固相萃取柱的选择

固定相的选择基本上是遵循相似相溶的原则,分析物的极性与固定相极性越相近,保留越好^[17]^。实验考察了C18 (200 mg/6 mL)、PLEXA(200 mg/6 mL)、Oasis HLB (200 mg/6 mL)和Oasis HLB (500 mg/6 mL) 4种固相萃取小柱对新烟碱类农药的萃取效果,如图2a。实验结果表明,C18萃取柱的萃取效率明显低于其他小柱,可能因为C18柱属于非极性固相萃取柱,而新烟碱类农药的极性较大,导致目标化合物回收率最高仅约70%;同样的原因,PLEXA萃取柱的填料是表面羟基化的聚苯乙烯-二乙烯基苯共聚物,适合中等极性到非极性化合物的萃取吸附,而呋虫胺由于极性过大导致PLEXA柱对呋虫胺的回收率仅40%左右;而HLB柱由*N*-乙烯吡咯烷酮和亲脂性的二乙烯基苯聚合而成的反相吸附剂组成,适用于极性化合物的萃取,因此HLB 200 mg和HLB 500 mg萃取柱对8种目标化合物的回收率均较高,分别为90.7%~93.3%和92.6%~95.7%。由于本研究对象是污水,而吸附剂量的增加可以提供更多的吸附位点^[18]^,因此选择HLB (500 mg/6 mL)固相萃取小柱。

**图2:**
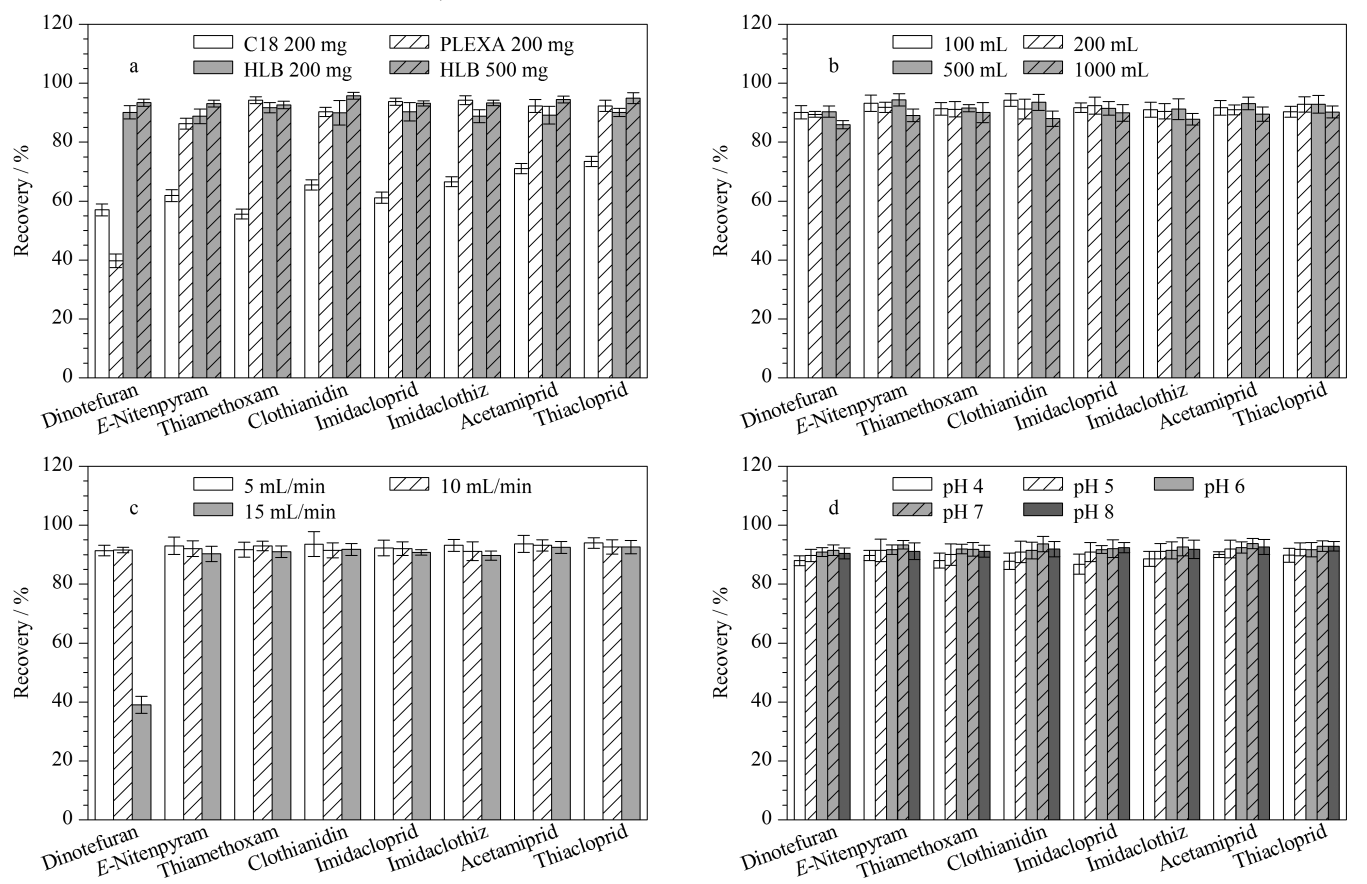
不同因素对目标化合物回收率的影响(*n*=3)

#### 2.2.2 上样体积的考察

理论上,增加样本体积可以提高检测的灵敏度,但实际操作中,样品体积过大会导致萃取柱中已活化延伸的碳链重新聚合,从而减少填料的表面积,降低萃取效率^[19]^。本文比较了上样体积分别为100、200、500和1000 mL时的萃取效率,结果如图2b所示。可以看出,上样体积为1000 mL时目标化合物回收率略低,而上样体积为100、200和500 mL时目标化合物回收率差别不大。考虑到样品的检测灵敏度,选取500 mL作为新烟碱类化合物的上样体积。

#### 2.2.3 上样速度的考察

上样速度影响固相萃取的吸附效率,以及吸附剂对样品中有机化合物的吸附能力,并直接影响前处理效率^[20]^。本实验比较了上样速度为5、10和15 mL/min时的萃取效率,结果如图2c所示。可以看出,上样速度为5 mL/min和10 mL/min时目标化合物回收率差别不大,而上样速度为15 mL/min时,呋虫胺的回收率低于40%。故选择上样速度为10 mL/min。

#### 2.2.4 水样pH值的选择

有研究表明,当pH值低于4.0时,新烟碱类杀虫剂带正电荷,静电排斥导致提取效率下降;而pH值高于8.0时,样品溶液中更多的阴离子OH^-^会与新烟碱类杀虫剂结合,导致提取效率下降^[21]^。本文考察了pH为4、5、6、7、8时新烟碱类农药的回收率(如图2d)。结果表明,不同酸度条件下新烟碱类农药的回收率差别不大,pH=4和pH=5时略低于其他水平。因此,在进一步的实验中,调节水样的pH为6~8。

### 2.3 基质效应的评估

Kebarle等^[10]^提出采用LC-MS/MS分析样品时,样品中除目标检测物之外的组分可能会增强或抑制目标物的信号强度^[22]^,这种现象为基质效应。Shah等^[9]^明确指出在LC-MS/MS方法的开发和验证过程中需要评估基质效应,以确保精度、选择性和灵敏度不会受到影响。提取后加标(PES)是评估基质效应的常用方法^[23]^。根据Matuszewski等^[24]^提出的方法,基质效应表示为空白样品基质提取后加标的峰面积与纯标准溶液的峰面积之比;回收率表示为空白样品基质提取前加标的峰面积与空白样品基质提取后加标的峰面积之比。即:


(1) $$ME=\frac{A_{A}-A_{NM}}{A_{s}}\times 100\%$$

(2) $$\text{ Recovery }=\frac{A_{B}-A_{NM}}{A_{A}-A_{NM}}\times 100\%$$

式中,*A*_s_是溶剂中的纯标准溶液的峰面积;*A*_A_是样品提取后加标的峰面积;*A*_B_是样品提取前加标的峰面积;*A*_NM_是样品非加标的峰面积。

当ME>100%表示电离增强,ME<100%表示电离抑制。|ME|为80%~120%时被认为是低基质效应;|ME|为50%~80%或120%~150%时被认为是中等基质效应;|ME|<50%或>150%时被认为是强基质效应^[12]^。

研究发现污水中基质效应较高,影响仪器定量,因此本文考察了优化色谱梯度洗脱程序、稀释进样及使用同位素内标定量降低基质效应的影响。

为避免基质组分与待测物同时洗脱,影响目标物的离子化效率,本文通过优化梯度洗脱程序来改变待测物的保留时间,使其远离离子抑制区域以降低基质效应的影响。当采用图3a的梯度洗脱条件对实际城镇污水中8种新烟碱类化合物进行全扫模式和多反应监测(MRM)扫描时,发现由于基质成分复杂,前两种化合物(呋虫胺和*E*-烯啶虫胺)出峰时间段基质对其影响较大,导致响应较小,且呋虫胺色谱峰形受影响。因此尝试减缓洗脱梯度,将原来的在3 min内将流动相中甲醇比例由10%增加到45%,优化为5 min(见图3b),延长了呋虫胺和*E*-烯啶虫胺的出峰时间,实现了其与复杂基质的分离,且优化后呋虫胺和*E*-烯啶虫胺响应值明显增加。

**图3:**
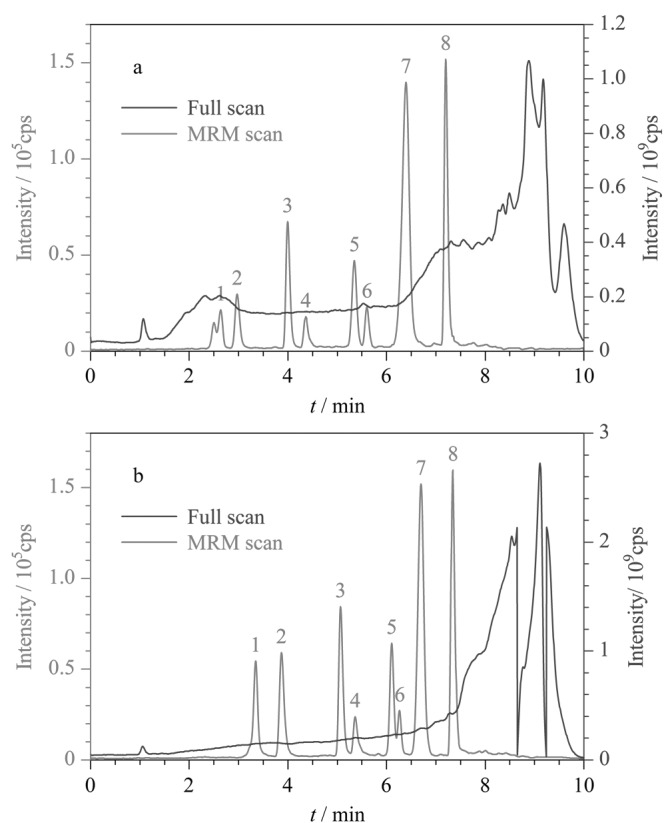
目标化合物在不同梯度洗脱程序下的色谱图

考察了稀释进样及使用同位素内标定量法对城镇污水中基质效应的影响,如图4所示,当污水不稀释上样500 mL且用外标法定量时,8种目标化合物的基质效应为16.6%~43.5%;而当稀释5倍和10倍进样,采用内标法定量时,基质效应为80%~120%,满足检测要求^[12]^。考虑到样品的检测灵敏度,选择取100 mL实际污水用纯水稀释5倍上样。

**图4:**
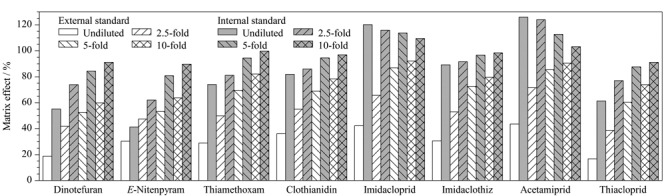
不同稀释倍数与定量方法对目标化合物基质效应的影响

### 2.4 响应曲面法优化

淋洗液、洗脱液和洗脱体积是去除污水中基质干扰和影响目标物回收率的关键因素。

为了去除基质干扰,可在洗脱前对固相萃取小柱进行淋洗。杨金泉等^[25]^在分析地表水中9种性激素时采用10%甲醇水溶液淋洗,在保证回收率的同时最大程度去除了基质干扰。因此初步确定淋洗液为甲醇水溶液。常用的洗脱液有二氯甲烷、丙酮、乙酸乙酯、甲醇、乙腈等^[26⇓⇓-29]^。二氯甲烷适用于非极性物质的洗脱^[30]^;丙酮和乙酸乙酯会溶解更多的杂质,影响样品的净化效果,并引起更强的基质效应^[31]^。因此本文考察甲醇、乙腈及其混合溶液作为洗脱液时的洗脱效果。对于洗脱体积而言,洗脱体积过小会导致目标化合物不能完全被洗脱,而过大的洗脱体积可能会把杂质洗脱下来,增加基质效应;同时过大的洗脱体积也会造成氮吹浓缩时间增加,导致目标化合物的损失。

因此本文在单因素优化和基质效应评估的基础上利用响应曲面法同时优化淋洗液甲醇水溶液中甲醇的体积分数(*X*_1_)、洗脱液甲醇-乙腈混合溶液中乙腈的体积分数(*X*_2_)和洗脱液的体积(*X*_3_)。每个变量的低、中、高3个级别指定为-1、0、+1,三因素与三水平见表2。使用Design Expert软件设计了17组实验,并对结果进行了图形分析和回归分析^[32]^,实验随机进行,以尽量减少非受控因素的影响^[33]^。

**表2:** 响应曲面的三因素与三水平

Factor	Code	Levels		
		-1	0	1
Volume fraction of methanol	*X*_1_	0	5	15
in water/%				
Volume fraction of acetonitrile	*X*_2_	0	50	100
in methanol/%				
Volume of eluent/mL	*X*_3_	3	6	9

方差分析统计检验(ANOVA)用于评估模型,*P*值通常用于检查变量的显著性,也可以反映每个独立变量之间的相互作用^[34]^。当*P*<0.05时,表示该因素是显著的模型项;当*P*>0.05时,表示模型项不显著。本研究以园区生产的噻虫啉作为代表进行分析,如表3所示,模型的*P*值为0.0001,说明该模型极其显著,同时预测模型与试验的拟合度用失拟项(Lack of fit)来表示,模型的失拟项*P*值为0.9903,相对于纯误差不显著,说明了该模型的适用性。模型的质量由模型拟合度(*R*^2^)决定,*R*^2^越接近1,说明模型和实际值之间具有更好的相关性^[35]^。经计算,*R*^2^为0.9710,调整后的*R*^2^ (Adjusted *R*^2^)、预测*R*^2^ (Predicted *R*^2^)分别为0.9338和0.9443,说明模型回归效果显著。在这种情况下,一次项*X*_2_、*X*_3_的*P*值均小于0.05,被视为显著项;其中,各因素对噻虫啉回收率的显著性顺序为*X*_3_*>X*_2_*>X*_1_。二次项 ${X_{2}}^{2}$ 、 ${X_{3}}^{2}$ 的*P*值均小于0.05,且两者系数均为负值,说明存在极大值点;在交互项中,*X*_1_*X*_2_、*X*_1_*X*_3_、*X*_2_*X*_3_对响应值的影响均不显著,说明各个因素对噻虫啉回收率的影响没有交互作用。信噪比15.45>4是理想的^[36]^。这些模型特征值表明了模型的可靠性,可用于确定噻虫啉回收率的最优条件。

**表3:** 回归模型方差分析

Source	Sum of square	Degree of freedom	Mean square	*F*- value	*P*- value
Model	288.37	9	32.04	26.06	0.0001^*^
*X*_1_	3.19	1	3.19	2.59	0.1514
*X*_2_	10.24	1	10.24	8.33	0.0235^*^
*X*_3_	112.5	1	112.5	91.5	<0.0001^*^
*X*_1_ *X*_2_	0.0025	1	0.0025	0.002	0.9653
*X*_1_ *X*_3_	0.0006	1	0.0006	0.0005	0.9826
*X*_2_ *X*_3_	0.0306	1	0.0306	0.0249	0.879
${X_{1}}^{2}$	0.005	1	0.005	0.0041	0.9509
${X_{2}}^{2}$	32.79	1	32.79	26.67	0.0013^*^
${X_{3}}^{2}$	121.78	1	121.78	99.05	<0.0001^*^
Residual	8.61	7	1.23		
Lack of fit	0.2144	3	0.0715	0.0341	0.9903
Pure error	8.39	4	2.1		
Cor total	296.98	16			

* Significant (*P*<0.05).

通过Design-Expert软件对噻虫啉数据结果进行多元二次回归拟合,得到*X*_1_、*X*_2_和*X*_3_ 3个变量对噻虫啉回收率(*Y*)的回归方程如下:


(3) *Y=*95*.*41*+*0*.*6313*X*_1_*-*1*.*13*X*_2_*+*3*.*75*X*_3_*-*0*.*025*X*_1_*X*_2_*-*0*.*0125*X*_1_*X*_3_*+*0*.*0875*X*_2_*X*_3_*+*0.0345X^2^_1_-2.79X^2^_2_-5.38X^2^_3_

三维响应曲面图及二维等高线平面图是二次多元非线性回归函数的图形表示形式。三维响应曲面图描述的是响应值相对于变量变化的敏感度差异,而二维等高线平面图描述的则是各变量之间的交互作用是否显著。等高线图呈椭圆或鞍形时表示相应变量之间的相互作用显著,圆形则相反^[37]^。如图5所示,通过对响应面图和等高线图的分析,响应面的形状呈凸形,有极值点,*X*_1_对噻虫啉回收率影响不显著,另外两个因素的相互作用都呈现先增加后降低的趋势。

**图5:**
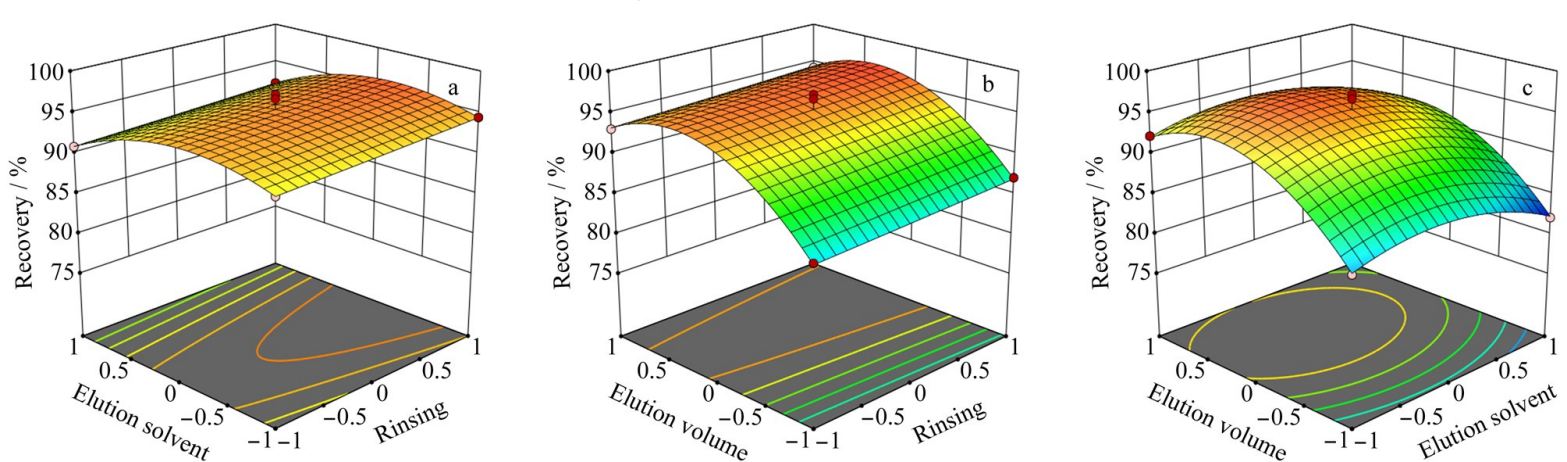
淋洗液、洗脱液和洗脱体积对噻虫啉回收率影响的响应曲面图

利用软件优化功能对式(3)进行求解,可得噻虫啉前处理方法的最佳参数如下:淋洗液为12.1%甲醇水溶液、洗脱液为甲醇-乙腈(7∶3, v/v)、洗脱体积为6.714 mL,此时噻虫啉的回收率为96.4%。对其余新烟碱类杀虫剂前处理最优条件进行逐个分析,结果如表4所示。可以看出,淋洗液中甲醇的体积分数为4.24%~13.32%,洗脱液中乙腈的体积分数为30%~80%,洗脱体积为6.444~7.137 mL,为确保8种新烟碱类农药的回收率都保持在较高水平及便于实际操作,选择最佳淋洗液为10%甲醇水溶液,洗脱液为甲醇-乙腈(1∶1, v/v),洗脱体积为7 mL。

**表4:** 8种新烟碱类农药通过响应曲面法获得的最优参数

Compound	*X*_1_/%	*X*_2_/%	*X*_3_/mL	Recovery/%
Dinotefuran	10.49	50	7.056	96.17
*E*-Nitenpyram	13.32	80	6.561	96.38
Thiamethoxam	9.32	50	6.444	96.63
Clothianidin	4.24	80	7.137	93.81
Imidacloprid	3.92	60	6.711	95.16
Imidaclothiz	6.08	30	6.678	96.01
Acetamiprid	6.66	60	7.026	98.53
Thiacloprid	12.10	70	6.714	96.47

### 2.5 方法学考察

#### 2.5.1 线性关系、检出限与定量限

配制8种新烟碱类农药系列标准工作溶液(其中同位素内标的质量浓度均为50 μg/L)。对待测物与内标物的峰面积比(*y*)与待测物的质量浓度(*x*, μg/L)进行线性回归,得出线性方程和相关系数(*r*)。按照HJ 168-2020中方法检出限(MDL)的要求,配制7个质量浓度为2 ng/L的样品进行固相萃取,对所得样品进行测量,计算7次平行测定的标准偏差(*S*), *t*(6, 0. 99)=3.143,则MDL=3.143*S*,方法定量限(MQL)为4倍MDL。结果如表5所示,8种新烟碱类化合物在各自线性范围内线性关系良好,*r*均大于0.999。MDL为0.2~1.2 ng/L, MQL为0.8~4.8 ng/L。

**表5:** 8种新烟碱类化合物的线性方程、相关系数、线性范围、方法检出限及方法定量限

Compound	Regression equation	*r*	Linear range/(μg/L)	MDL/(ng/L)	MQL/(ng/L)
Dinotefuran	*y*=1.723*x*+0.0374	0.9996	0.5-100	0.8	3.2
*E*-Nitenpyram	*y*=1.545*x*+0.0376	0.9995	0.2-100	0.4	1.6
Thiamethoxam	*y*=3.734*x*+0.0654	0.9996	0.2-100	0.5	2.0
Clothianidin	*y*=0.499*x*+0.013	0.9991	0.2-100	0.6	2.4
Imidacloprid	*y*=1.345*x*+0.0458	0.9995	0.5-100	1.2	4.8
Imidaclothiz	*y*=1.302*x*+0.0256	0.9993	0.2-100	0.4	1.6
Acetamiprid	*y*=7.443*x*+0.201	0.9994	0.1-100	0.2	0.8
Thiacloprid	*y*=7.342*x*+0.091	0.9995	0.1-100	0.3	1.2

*y*: ratio of peak areas of analyte to internal standard; *x*: mass concentration, μg/L.

#### 2.5.2 回收率和精密度

配制低(10 ng/L)、中(200 ng/L)、高(1000 ng/L)3个水平的空白加标样各3份,按照1.2节和1.3节方法进行实验,计算加标回收率和相对标准偏差(RSD),结果如表6所示,在不同加标水平下,8种新烟碱类化合物的加标回收率为82.6%~94.2%, RSD为3.9%~9.4%。

**表6:** 8种新烟碱类化合物在3个水平下的加标回收率和RSD (*n*=3)

Compound	Added/(ng/L)	Recovery/%	RSD/%	Compound	Added/(ng/L)	Recovery/%	RSD/%
Dinotefuran	10	86.4	8.7	Imidacloprid	10	87.1	9.4
	200	93.8	6.5		200	91.8	5.9
	1000	94.2	7.9		1000	87.5	5.5
*E*-Nitenpyram	10	91.8	7.9	Imidaclothiz	10	85.4	8.6
	200	93.5	6.5		200	92.5	7.8
	1000	93.8	5.8		1000	91.7	6.9
Thiamethoxam	10	89.4	9.4	Acetamiprid	10	82.6	6.8
	200	92.1	8.4		200	86.8	4.7
	1000	94.1	5.2		1000	92.7	6.2
Clothianidin	10	87.2	9.3	Thiacloprid	10	85.8	6.4
	200	92.1	5.8		200	91.1	4.5
	1000	93.3	6.7		1000	93.4	3.9

#### 2.5.3 与文献方法比较

将该方法与类似方法进行比较,如表7所示,该方法线性范围宽,正确度高,精密度好,方法检出限低于目前文献中污水基质,同清洁水体中的方法检出限相当。

**表7:** 与其他文献的比较

Analytes	Sample type	Sample volume/ (mL)	MDL/ (ng/L)		Linear range/ (μg/L)		RSD/%		RE/%	Ref.	
Dinotefuran, *E*-nitenpyram, thiamethoxam, clothianidin, imidacloprid, imidaclothiz, acetamiprid, thiacloprid	wastewater	100	0.2-	1.2	0.1-	100^*^	3.9-	9.4	82.6-94.2	this study	
Acetamiprid, imidacloprid, thiacloprid, thiamethoxam, clothianidin, dinotefuran	wastewater	500	1.8-	6.8	1-	500	5.26-	11.5	33.7-116	[17]	
Dinotefuran, thiamethoxam, clothianidin, imidacloprid, imidaclothiz, acetamiprid, thiacloprid	drinking water	500	0.01-	0.2	0.01-	200	1.6-	7.3	74.0-123	[9]	
Thiamethoxam, clothianidin, imidacloprid, acetamiprid,	sea water	1000	0.1-	7.8	0.05-	100	3-	18	72.0-117	[24]	
thiacloprid	river water		0.1-	1.0							

RE: recovery. * Details were shown in Table 5.

### 2.6 实际水样检测

应用本方法对4个城镇污水处理厂的进水水样(S1、S2、S3、S4)进行分析,如表8所示,8种新烟碱类农药检出含量为ND~256 ng/L,基质效应为80.9%~113.7%,对分析复杂环境水体中新烟碱类农药具有适用性。

**表8:** 实际废水样品中8种新烟碱类化合物的含量和基质效应

Compound	Contents/(ng/L)				ME/%
	S1	S2	S3	S4	
Dinotefuran	ND	5.49	6.69	23.1	84.3-98.7
*E*-Nitenpyram	ND	4.83	5.39	13.2	80.9-96.2
Thiamethoxam	11.6	18.1	9.46	15.2	90.4-103.5
Clothianidin	8.91	13.4	10.7	17.9	89.7-108.1
Imidacloprid	31.1	256	178	189	97.2-113.7
Imidaclothiz	ND	14.5	14.2	12.3	93.2-104.8
Acetamiprid	8.45	17.9	16.6	57.2	96.9-112.7
Thiacloprid	135	1.98	2.47	1.89	87.6-105.3

ND: not detected.

## 3 结论

本工作建立了SPE-LC-MS/MS测定城镇污水中8种新烟碱类农药的方法。通过单因素实验和响应面法优化了前处理条件,并通过用优化色谱-质谱条件、稀释进样、同位素内标法定量等,降低了基质效应对测定结果的影响。该方法适用于城镇污水中痕量新烟碱农药的检测,可为监管部门的管理提供技术支撑。
